# Aerobes and phototrophs as microbial organic fertilizers: Exploring mineralization, fertilization and plant protection features

**DOI:** 10.1371/journal.pone.0262497

**Published:** 2022-02-02

**Authors:** Eva Wambacq, Abbas Alloul, Oliver Grunert, Jasper Carrette, Pieter Vermeir, Janne Spanoghe, Myrsini Sakarika, Siegfried E. Vlaeminck, Geert Haesaert

**Affiliations:** 1 Department of Plants and Crops, Faculty of Bioscience Engineering, Ghent University, Ghent, Belgium; 2 Research Centre AgroFoodNature, School of Bioscience and Industrial Technology, University of Applied Sciences and Arts, Gent, Belgium; 3 Department of Bioscience Engineering, Research Group of Sustainable Energy, Air and Water Technology, Faculty of Science, University of Antwerp, Antwerpen, Belgium; 4 Greenyard Horticulture Belgium, Gent, Belgium; 5 Department of Green Chemistry and Technology, Faculty of Bioscience Engineering, Ghent University, Gent, Belgium; Soil and Water Resources Institute ELGO-DIMITRA, GREECE

## Abstract

Organic fertilizers and especially microbial biomass, also known as microbial fertilizer, can enable a paradigm shift to the conventional fertilizer-to-food chain, particularly when produced on secondary resources. Microbial fertilizers are already common practice (e.g. Bloom^®^ and Synagro); yet microbial fertilizer blends to align the nutrient release profile to the plant’s needs are, thus far, unexplored. Moreover, most research only focuses on direct fertilization effects without considering added value properties, such as disease prevention. This study has explored three promising types of microbial fertilizers, namely dried biomass from a consortium of aerobic heterotrophic bacteria, a microalga (*Arthrospira platensis*) and a purple non-sulfur bacterium (*Rhodobacter sphaeroides*). Mineralization and nitrification experiments showed that the nitrogen mineralization profile can be tuned to the plant’s needs by blending microbial fertilizers, without having toxic ammonium peaks. In a pot trial with perennial ryegrass (*Lolium perenne* L.), the performance of microbial fertilizers was similar to the reference organic fertilizer, with cumulative dry matter yields of 5.6–6.7 g per pot. This was confirmed in a pot trial with tomato (*Solanum lycopersicum* L.), showing an average total plant length of 90–99 cm after a growing period of 62 days for the reference organic fertilizer and the microbial fertilizers. Moreover, tomato plants artificially infected with powdery mildew (*Oidium neolycopersici*), a devastating disease for the horticultural industry, showed reduced disease symptoms when *A*. *platensis* was present in the growing medium. These findings strengthen the application potential of this novel class of organic fertilizers in the bioeconomy, with a promising match between nutrient mineralization and plant requirements as well as added value in crop protection.

## Introduction

Our industrialized global society is heavily dependent on synthetic inorganic fertilizers for primary crop production in both agriculture and horticulture. It is estimated that over 110 million tons of N fertilizer (e.g. ammonium nitrate, ammonium sulfate and urea), 10.3 million tons of P and 15.6 million tons of K are annually consumed [1, 2]. Intensive fertilizer use has, however, serious environmental and economic repercussions [3]. When fertilizers are applied to the soil or in growing media (GM) for agricultural and horticultural applications, they are not completely consumed by plants, yet suffer from inefficiencies such as leaching, runoff and volatilization. For every 100 units of fertilizer applied to the land, only 4–14 units of nitrogen and 17 units of phosphorus are eventually consumed by humans [4–6]. A considerable portion of these fertilizers ultimately end up in the environment, resulting in detrimental effects on water quality (e.g. eutrophication), air quality (e.g. emissions of ammonia and nitrous oxide), the greenhouse gas balance (e.g. nitrous oxide), biodiversity and soil quality (e.g. acidification of soils) [7, 8].

Recycling nutrients from secondary resources may offer an improvement to the overall efficiency of the fertilizer-to-food chain [9]. Solid and liquid by-products and residues from plant and animal origin, such as animal manures, animal slurries, blood meal, cocoa shells, soybean meal and organic waste from restaurants and supermarkets play a central role [10]. These so-called organic fertilizers slowly release nutrients through decomposition or decay imposed by the microbiome present in the growing medium or the soil [11]. Organic fertilizers may provide benefits for the soil, such as better water retention, improved nutrient retention and an increase in the organic matter content of the soil, which on its account buffers the soil against salinity, pH changes and pesticides [12–15]. These organic fertilizers contribute to 5% of the total fertilizer market, yet their share will become more significant, as their annual growth rate was around 14% in 2019 compared to only 4% for the total fertilizer market [16].

Microbial fertilizers constitute a novel and promising class of organic fertilizers based on using microbial biomass as a source of plant nutrients [17, 18]. The microbes can be produced on secondary resources such as industrial wastewater, sewage, manure, etc [19, 20]. Microbial biomass can serve as a multi-nutrient fertilizer mainly rich in nitrogen (7–9 g N 100 g^-1^ dry matter) with an elemental composition of C_4.2_O_1.8_H_0.8_NP_0.2_S_0.1_K_0.1_ [18, 21]. In principle, dried microbial biomass produced on secondary resources is mixed with a growing medium or soil [17, 18]. The microbiome present in the growing medium or soil will then mineralize the microbial biomass, thereby making nutrients available for plant growth [11].

Three types of microbial fertilizers are generally considered for microbial fertilizer production on secondary resources: (i) a consortium of aerobic heterotrophic bacteria (AHB); [22], (ii) photoautotrophic microalgae (MA); [23, 24] and (iii) photoheterotrophic purple non-sulfur bacteria (PNSB); [17, 18, 25, 26]. AHB are probably the most widely applied and explored microbial fertilizers. A very common type of AHB is treated and stabilized sewage sludge a.k.a. biosolids (Bloom® and Synagro). In Europe, for example, around 10 million tons dry matter is annually produced [27]. Extensive research has been performed on the mineralization and fertilization properties of biosolids. A review of 32 studies by Rigby, Clarke showed that the mineralizable nitrogen decreased with increasing biological stabilization [28]. In terms of fertilization, increased crop yields have been reported for different plants such as rice, radish, wheat and barley [29]. Added value properties such as plant protection have, according to the authors’ knowledge, not yet been reported.

MA have also been explored as a microbial fertilizer, yet not as extensively as AHB. The mineralization of different types of MA has been studied, showing different final plant-available N fractions for *Nannochloropsis* biomass (31% after 95 days); [24], microalgal bacterial flocs (25% after 95 days); [8, 30], *Arthrospira platensis* biomass (72% N after 77 days); Spanoghe, Grunert (18) and algal biomass grown on manure effluents (41% after 63 days). In terms of fertilization, several plants have been explored such as cucumber and cord seedlings, parsley (*Petroselinum crispum*), petunia and tomato (*Solanum lycopersicum* L.), showing an equal or improved performance compared to commercial inorganic and/or organic fertilizers [8, 18, 24, 30]. Relatively to AHB, added value properties of MA and their extracts have extensively been reported in terms of (a)biotic plant protection (e.g. antifungal, bacterial and antinematodal activity, alleviation of drought and salt stress) and biostimulation (e.g. increase in germination rate, salt tolerance, nutritional value, etc.) [31].

PNSB are probably the most novel type of microbial fertilizer. Mineralization studies on PNSB biomass are, however, limited to our previous research on *Rhodobacter sphaeroides*. A final plant-available N fraction of 70% was observed after 77 days [18]. Also, as fertilizer, only a few plants were tested such as pasture ryegrass (*Lolium rigidum Gaudin*), mandarine tree (*Citrus reticulata*) and parsley (*Petroselinum crispum*) [18, 20, 32]. Nonetheless, all results showed a positive or comparable effect of PNSB biomass on plant growth or fruit quality and production relative to the control [18, 20, 32]. In terms of added value properties of PNSB for plants, multiple researchers have reported growth promotion and alleviation of environmental stress [17].

Although AHB, MA and PNSB have been studied as a source of microbial fertilizers, research is mainly limited to their individual contribution to plant growth. Our previous research is one of the first to study blends and indicated that it is economically sensible because it can provide cheap nutrients and added value for plants [18]. The goal of this study was to gain a broader understanding of the applicability across horticultural and agricultural for microbial fertilizers based on AHB, MA and PNSB, both individually as well as in blends. First, nitrogen mineralization and nitrification profiles were determined in a commercially relevant growing medium. Second, the fertilization effect of the microbial fertilizers was evaluated on the plant growth performance in pot trials with perennial ryegrass (*Lolium perenne* L.) and tomato. Third, disease susceptibility towards a biotrophic fungus (*Oidium neolycopersici*) and two necrotrophic fungi (i.e. *Alternaria solani* and *A*. *alternata*) was assessed for tomato, to explore added value properties of the three microbial fertilizers.

## Materials and methods

### Growing medium

A non-sterilized, nutrient-poor organic growing medium (produced by Greenyard Horticulture), consisting of 20 vol. % black peat, 50 vol. % white peat, 20 vol. % coco coir pith and 10 vol. % green waste compost (RHP certified), was used for all experiments. The pH was adjusted to 5.5–6.0 by adding lime (Ca,Mg(CO_3_)_2_) at 3 kg m^-^³ with an acid-binding capacity of 55% to increase the availability of trace elements such as Zn, Cu and Fe. The exact growing medium composition is given in S1 Table. Electrical conductivity (EN 13038) and pH (EN 13037) were measured in a 1:5 soil to water (v/v) suspension. Water-soluble nutrients and elements (i.e. NO_3_^-^, NH_4_^+^, Cl^-^, Na^+^, SO_4_^2-^ and PO_4_^3-^) were extracted (1:5 v/v) according to EN 13652, and measured with a Dionex DX-600 IC ion chromatography (Dionex, Sunnyvale, CA), and for ammonium with a Skalar San++ mineral nitrogen analyzer. Plant-available concentrations of P, K, Ca, Mg, Fe and Mn were extracted (1:5 v/v) in ammonium acetate buffered at pH 4.65, and measured by CCD simultaneous ICP-OES (VISTA-PRO, Varian, Palo Alto, CA). Physical characteristics of the growing medium were measured according to EN 13041 (DIN 2012). Fresh bulk density of each batch of non-compacted growing medium was determined according to EN 12580.

### Microbial fertilizers

Three microbial fertilizers were tested, all as dried powder. AHB were produced by Avecom (Wondelgem, Belgium) on a nutrient-rich side stream from a potato processing company on a semi-industrial scale. No trace elements were administered in the reactor. The MA *Arthrospira platensis* (Spirulina), was cultured by AgrAqua (Oosterzele, Belgium) in a semi-industrial raceway pond in a foil greenhouse tunnel on cutting process water from a potato processing company, complemented with struvite [33] and concentrated discharge water of an organic acid air scrubber (pretreated with a lava filter for nitrification). The broth was supplemented with iron chelate, sodium carbonate and sodium bicarbonate [34]. No trace elements were administered. *Rhodobacter sphaeroides* was selected as PNSB microbial fertilizer based on its high growth performance in a previous study [25]. PNSB were cultured on synthetic wastewater containing a 1/1/1 mixture of acetic acid, butyric acid and propionic acid on carbon basis at lab-scale in the laboratories of the University of Antwerp (Antwerp, Belgium) [35]. A detailed description of the microbial fertilizers production can be found in our previous paper [18].

The three types of microbial fertilizers were investigated individually and in blends (Table 1). Blends were tested because microbial fertilizers solely based on phototrophs (MA or PNSB) are economically less attractive. In a previous cost estimation, AHB/MA or AHB/PNSB blends of 85%/15% showed to be more cost-competitive [18]. The ratios of the blended microbial fertilizers, therefore, contained a higher amount of AHB (75–95%) compared to MA and PNSB (5–25%).

**Table 1:** Experimental treatments in the three experiments. Fertilizer blend ratios are expressed according to nitrogen supply. The numbers in the treatment codes refers to the blend ratio expressed in % dry matter. No fertilizers were added for the negative control experiment. The species A*rthrospira platensis* and *Rhodobacter sphaeroides* were used for respectively the microalga (MA) and purple non-sulfur bacteria (PNSB) fertilizers. Two ryegrass pot trials were performed with either individual (a) or microbial fertilizer blends (b). AHB: aerobic heterotrophic bacteria; SF2: conventional organic fertilizer.

Treatment Code	N mineralization	Ryegrass pot trials		Tomato pot trial
		a	b	
Neg. control	x	x	x	x
SF2	x	x	x	x
AHB	x	x		x
MA	x	x		x
PNSB	x			x
AHB+MA 85/15	x		x	x
AHB+PNSB 85/15	x		x	x
AHB+MA+PNSB 85/7.5/7.5	x		x	x
AHB+MA 75/25				x*
AHB+PNSB 75/25				x*
AHB+MA+PNSB 75/12.5/12.5				x*
AHB+MA 95/5				x*
AHB+PNSB 95/5				x*

* These treatments were not included in the *Alternaria* leaf spot assay of the tomato trial.

### Fertilizer mineralization and nitrification

The nitrogen mineralization profiling experiment had the objective to characterize the release pattern of ammonium and nitrate for the individual and blended microbial fertilizers incorporated into the growing medium.

A batch incubation test was performed to determine the *in vitro* nitrogen mineralization profile of the microbial fertilizers compared to a negative control (i.e. growing medium not supplemented with any fertilizer) and a conventional organic fertilizer provided by Greenyard Horticulture (SF2; Frayssinet, France). SF2 is made of fruit cake and pulp, composted poultry manure, processed animal proteins (containing hydrolyzed feathers, bone and meat powders, horn powder and dried blood), and vinasses (conform regulation EC 1069/2009). Total nitrogen (EN 13654–2 VarioMax Elementar), phosphorus (EC 2003/2003 BNL-P-2 and BNL-K-1, Interpid 2 XSP (Thermo) spectrometer) and potassium were determined before the experiment for all individual fertilizers (Table 2). The fertilizers were, then, dosed to the growing medium (bulk density 351 g L^-1^) to supply 352 mg-N L^-1^. Per treatment, fifteen 6 x 6 x 6 cm pots were filled with 0.205 L of growing medium. Three pots per treatment were immediately sampled (i.e. day 0), while the remaining pots were placed in a randomized block arrangement in a climate chamber at 22°C. Sampling was performed after 7, 14, 28 and 42 days (N = 3). Dry matter (DM) content of the growing medium was determined according to CMA/2/IV/1. A watery extract of the growing medium was prepared according to CMA/2/IV/24 and CMA/2/IV/4) and ammonia-nitrogen, nitrate-nitrogen (CMA/2/IV/7) and pH (CMA/2/IV/13) were quantified. The ammonia-nitrogen and nitrate-nitrogen levels of the negative control at the start of the experiment (day 0) were subtracted from the levels detected for the other treatments at all sampling moments.

**Table 2:** NPK content of fertilizers: Conventional organic fertilizer SF2, aerobic heterotrophic bacteria (AHB), *Arthrospira platensis* as microalga (MA) and *Rhodobacter sphaeroides* as purple non-sulfur bacteria (PNSB).

Fertilizer	N	P_2_O_5_	K_2_O
	(g kg^-1^)	(g kg^-1^)	(g kg^-1^)
SF2	80	43	65
AHB	70	34	48
MA	86	14	17
PNSB	58	110	11

### Fertilization pot trial with perennial ryegrass

The perennial ryegrass pot trial had the objective to evaluate the effect of the microbial fertilizers on plant growth (i.e. dry matter production). Due to the limited availability of the microbial fertilizers, two pot trials were conducted with perennial ryegrass cv. ‘Plenty’. Both trials included a negative (no fertilizer) and positive control (i.e. reference organic fertilizer SF2). The experiments aim to provide all nitrogen through microbial fertilizers. The fertilizers were, therefore, dosed to supply 421 mg-N L^-1^ growing medium and were blended in the growing medium (bulk density 351 g L^-1^) at the start of the experiment. No additional phosphorus and potassium were added because it was not a practical orientated test (vs. tomato pot trial). The key objective was to study direct effects of the microbial fertilizers. The first trial focused on individual fertilizers and the second trial tested microbial fertilizers blends (details Table 1).

The experimental setup was similar for both pot trials. For every treatment, five pots of 11 x 11 x 12 cm (length x width x height) were filled with 1 liter of the growing medium/fertilizer mixture (N = 5). On top of the mixtures, one gram of grass seeds was spread per pot. Pots were placed in a growing chamber at 22°C, 80% relative humidity (RH) with a 12 h light/12h dark light regime in a randomized block arrangement. Pots were individually sub-irrigated with tap water (electrical conductivity = 0.35 mS cm^-1^). There were no additional nitrogen or other compounds supplemented during the 160 days growing period. Nine cuts of grass were harvested by manual cutting. Fresh matter and dry matter (determined by air drying at 65°C) were determined for each cut, and cumulative aboveground dry matter yield was calculated.

### Fertilization and plant protection pot trial with tomato

The aim of the tomato pot trial was to study the effect of microbial fertilizers on plant growth and explore induced tolerance towards plant-pathogenic fungi.

**Fig 1:**
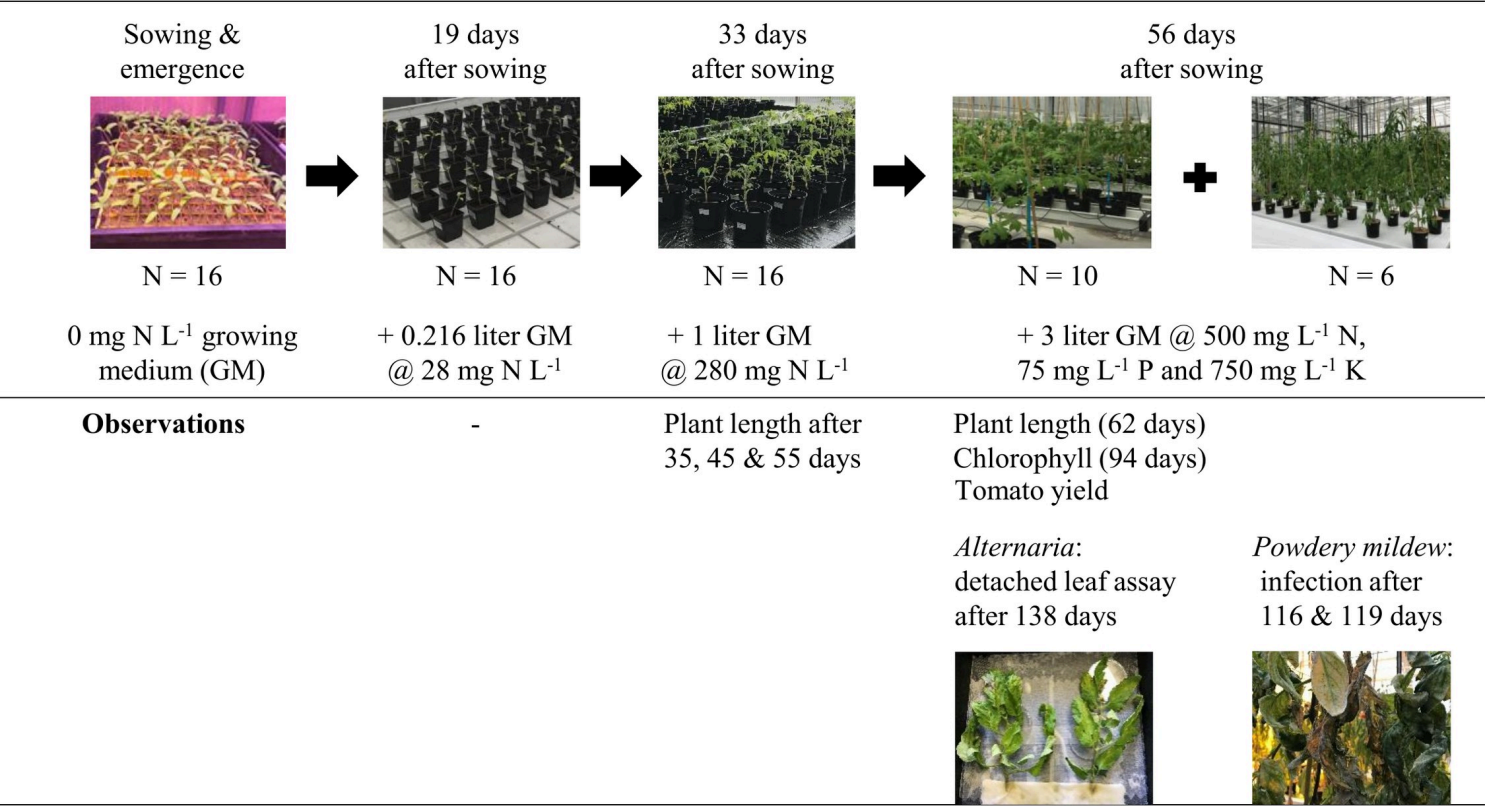
Schematic overview of the experimental setup of the pot trial with tomato.

The pot trial with tomato cv. ‘Pyros’ was conducted from May 19^th^, 2017 to Oct 16^th^, 2017. A combination of three individual and eight microbial fertilizer blends were tested (Table 1). The experimental setup of this pot trial with tomato is visualized in Fig 1. Seeds were, first, sown in growing medium not supplemented with nitrogen (bulk density 307 g L^-1^) in trays and incubated in a growing chamber at 22°C, 80% RH and 12 h light/12h dark light regime. After 19 days, the seedlings (incl. clod) were transferred to 6 x 6 x 6 cm pots with 0.216 liter of growing medium containing 28 mg-N L^-1^ (N = 16 + 3 as backup) with the appropriate fertilizers blended in the growing medium. Fifteen days later, the plants (incl. clod) were transferred again to round 1-liter pots containing 1 liter of growing medium supplemented with 280 mg-N L^-1^ according to the treatments (fertilizers blended in the growing medium). Both the 6 x 6 x 6 cm pots and 1-liter pots were placed on ebb-and-flow benches (sub-irrigation with mixed water of electrical conductivity = 0.40 mS cm^-1^) in an automatically ventilated glass greenhouse (day temperature 22°C and night temperature 18°C) of Ghent University (50°59’36.6” N and 3°47’05.1” E) without additional lightening according to a randomized block arrangement. After a growing period of 56 days, the plants (incl. clod) were transferred one last time, to round 4-liter pots with 3 liter of growing medium containing 500 mg of nitrogen per liter, according to the treatments (fertilizers blended in the growing medium). To ensure an excess of phosphorus and potassium, 75 mg-P L^-1^ growing medium (as triple superphosphate) and 750 mg-K L^-1^ growing medium (as potassium sulfate) were supplemented to all treatments. The N-P-K ratio provided by the fertilizer was 1 g-N/0.2–0.4 g-P/1.8–2.0 g-K and fully covered the plant’s needs (1 g-N/0.03 g-P/0.36 g-K) [36]. Side shoots were removed regularly throughout the pot trial, and plants were stopped at two leaves above the second inflorescence.

Ten replicates per treatment were placed *ad random* in gutters (inclination 0°16’2”) and irrigated with individual drippers (providing water of electrical conductivity = 0.40 mS cm^-1^). Six remaining replicates per treatment were placed on ebb-and-flow benches (sub-irrigation with mixed water of electrical conductivity = 0.4 mS cm^-1^) in a quarantine greenhouse (day temperature 22°C, night temperature 18°C, RH > 70%) and arranged in a randomized block design. Freshly collected powdery mildew (*Oidium neolycopersici*) from a tomato greenhouse in Kruishoutem (50°54’16.99” N and 3°31’41.02” E) was used to infect the plants. An *O*. *neolycopersici* spore suspension was used to artificially infect the plants after 116 (2 liter at 1.10^5^ spores mL^-1^) and 119 (1.5 liter at 4.10^4^ spores mL^-1^) days of growth by vaporizing. Powdery mildew infestation on the plants was scored at 22 days after the first infection according to the following scale: 0 = no infection, 0.5 = limited infection, 1 = heavy infection. The negative control could not be scored due to severe growth retardation. The average score per treatment represents the disease index.

Plant length was registered at 35, 45, 55 and 62 days after sowing for the ten plants per treatment kept in gutters (not-infected). The leaf greenness index of the leaf (n = 10) just below the first inflorescence was determined at 94 days after sowing (Minolta SPAD-502, self-calibrating, five readings per plant). Five leaves were taken after 138 days from all treatments for a detached-leaf assay to evaluate the sensitivity towards *Alternaria* leaf spot (21, 22). The microbial fertilizers blends containing 75% and 95% of the nitrogen supplied by AHB were not sampled because it was logistically impossible to include all treatments. On three sites per leaf, a 10-μl droplet of spore suspension (containing 0.5*10^5^ spores of isolates *A*. *alternata* 14.20+ and *A*. *solani* 15.2 mL^-1^ (isolates from the collection of the Experimental Farm Bottelare, multiplied on potato dextrose agar medium for 10 days at 20°C), supplemented with 5 gram of Potato Dextrose Broth per liter) was brought. Leaf spot-infested surface area was registered after 12 days of incubation in the dark at 100% RH and 20°C. An overview of this trial is presented in Fig 1.

### Statistical analyses

The obtained data were statistically analyzed with SPSS 25 software. Significance was declared at 95%. Normality was checked by Shapiro-Wilk’s test (applying Bonferroni correction) and homogeneity of variances was checked with Levene’s test. When both conditions were met, a one-way ANOVA with Tukey as *post hoc* test was performed. In case of normal distribution but no homogeneity of variances, a Welch’ Anova was performed with Dunnett T3 as *post hoc* test. Parameters lacking a normal distribution were subjected to non-parametric testing according to Kruskal-Wallis, with Dunn’s test for pairwise comparisons (applying Bonferroni correction).

## Results and discussion

### Fertilizer mineralization and nitrification

**Fig 2:**
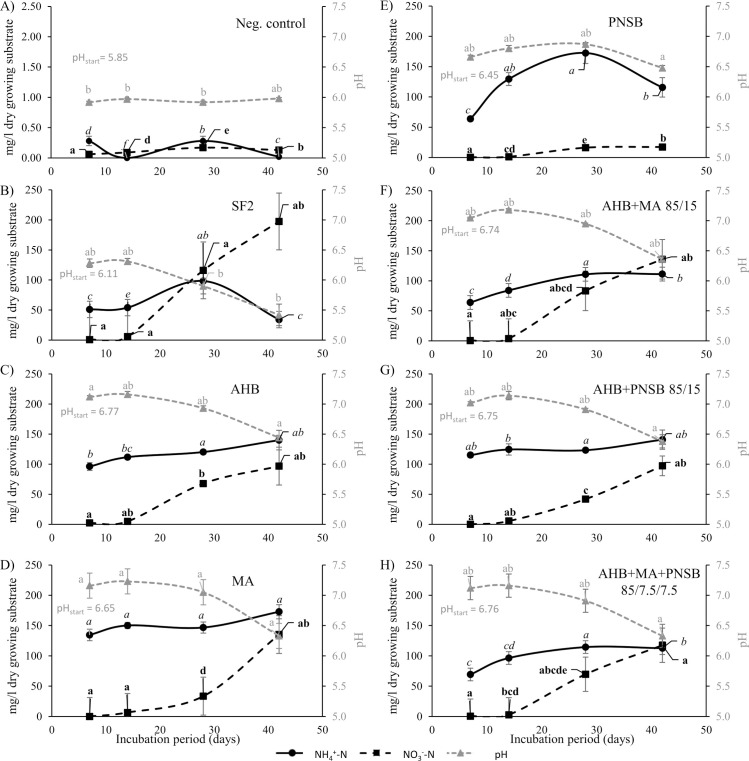
Nitrogen mineralization and nitrification: Profiles of total ammoniacal nitrogen, nitrate nitrogen and pH. (A) organic growing medium without fertilizer (different primary y-axis), (B) conventional organic fertilizer SF2, (C) aerobic heterotrophic bacteria (AHB), (D) microalga (MA), (E) purple non-sulfur bacterium (PNSB), (F) 85% AHB and 15% MA, (G) 85% AHB and 15% PNSB and (H) 85% AHB, 12.5% MA and 12.5% PNSB. All individual or fertilizers blends were supplied at 352 mg-N L^-1^. Error bars represent the standard deviations. Significant differences among treatments are indicated per sampling point by letter code for ammonia nitrogen, nitrate nitrogen and pH, resp. in italic, bold and grey.

During this experiment, the release pattern of ammonium and nitrate for the individual and blended microbial fertilizers was characterized (Fig 2). Minor nitrogen mineralization was observed in the negative control, whereas divergent mineralization patterns were found for the different fertilizer treatments. The conventional organic fertilizer SF2 was characterized by relatively low ammonium levels and rapidly increasing nitrate levels. The AHB fertilizer triggered a gradual release of both ammonium and nitrate, as did the blends containing this microbial fertilizer. MA showed high and almost unchanging ammonium concentrations, while the nitrate concentration showed a greater increase with passing incubation period. PNSB produced minor nitrate concentrations, while ammonium concentrations were ascending steadily during the first 30 days of the incubation period and descending behavior thereafter.

Mineralization started after an incubation period of 14 days. After 42 days, biological conversion of organic nitrogen to ammonium and nitrate was approximately 88% for MA, around 68% for SF2, AHB, AHB+MA 85/15 and AHB+PNSB 85/15, around 62% for the blend AHB+MA+PNSB 85/7.5/7.5 and 34% for PNSB. The final ammonium to nitrate ratio for the conventional organic fertilizer was 1:5, while AHB, MA, PNSB, and the three blends had a final ammonium to nitrate ratio of respectively 1:0.6, 1:0.6, 1:0.8, 1:0.2, 1:1.4, 1:0.7 and 1:1. Research indicates that the highest plant yields are achieved by a combined supply of both ammonium and nitrate [37, 38]. Crops may be classified into four types: (i) preference to ammonium; (ii) preferences to nitrate; (iii) equal effect of ammonium and nitrate; (iv) combinative use of the two nitrogen sources being superior to either ammonium or nitrate alone. Recent results indicate that aerobes, phototrophs and combinations thereof could be used as organic fertilizer for plants with different nutrient requirements and nitrogen preferences [39].

pH differences in the growing medium/fertilizer mixtures were minor and did not show an effect over time. The organic fertilizers SF2, on the other hand, had a pH-lowering effect. When organic nitrogen is converted to ammonia it directly withdraws an H^+^ to form ammonium, so an OH^-^ remains and triggers an increase of the pH of the growing medium, as observed during the first 14 days of the experiment. The pH decline for SF2 during days 28–42 was due to microbial conversion of ammonium to nitrate (196 mg NO_3_^—^N/L produced, see Fig 2).

### Fertilization pot trial with perennial ryegrass

**Fig 3:**
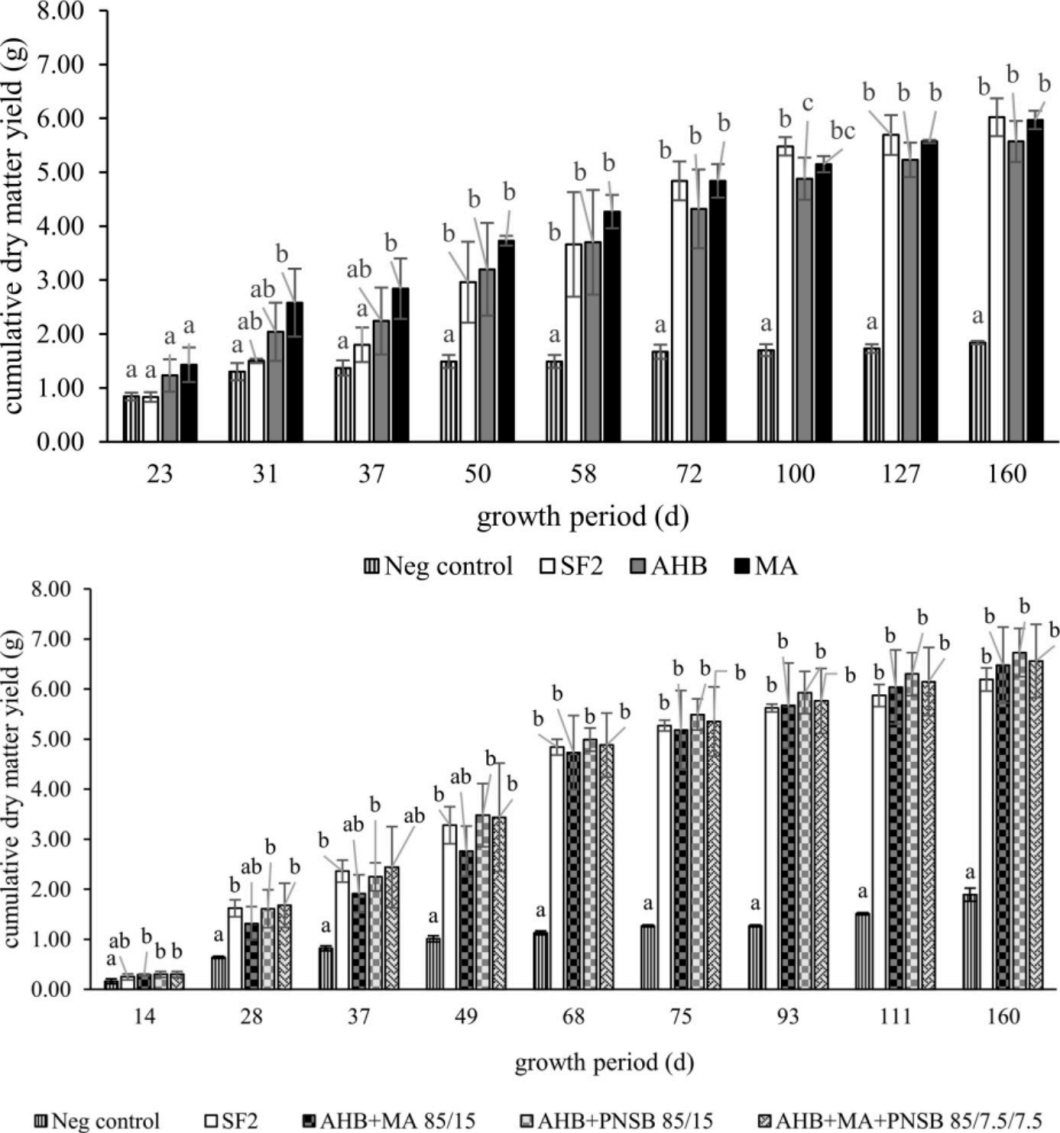
Perennial ryegrass: Cumulative aboveground dry matter (DM) yield. A non-fertilized negative control (Neg control) was compared to a conventional organic fertilizer (SF2) and three microbial fertilizers, i.e. aerobic heterotrophic bacteria (AHB), microalga (MA) and purple non-sulfur bacteria (PNSB) individually or in blends (according to nitrogen supply). Significant differences among treatments are indicated by letter code per sampling point, while error bars represent the standard deviation.

Perennial ryegrass pot trials were performed to study the effect of the microbial fertilizers on plant growth. The cumulative aboveground dry matter yields of all fertilized treatments outperformed the negative control in both pot trials from the fourth and the fifth cut onwards (Fig 3). The microbial fertilizers performed equally well as the conventional organic fertilizer SF2. No ammonium toxicity symptoms were detected. A similar performance was reported for an enriched PNSB biomass (*Rhodopseudomonas* sp.) produced on pig farm wastewater in a pot trial with pasture ryegrass (*Lolium rigidum Gaudin*), showing an equal performance in terms of shoot dry weight compared to an inorganic fertilizer. MA biomass composed of *Chlorella* sp. and *Scenedesmus* sp., on the other hand, resulted in a lower shoot dry weight than the inorganic fertilizers.

Plants have an impact on their growing medium by exudation of organic substances in the rhizosphere, influencing microbial community composition and activity, nutrient cycling and pH [40]. Therefore, the nitrogen mineralization pattern observed in crop-free growing medium can differ somewhat from the pattern observed in practice, when plants are grown in the growing medium. Regarding plant performance, the ryegrass pot trials demonstrated that the microbial fertilizers performed equally well as the reference organic fertilizer in aboveground dry matter production.

### Fertilization and plant protection pot trial with tomato

The tomato pot trial was performed to study the effect of individual and blends of microbial fertilizers on plant growth and explore tolerance towards plant-pathogenic fungi.

Plant lengths are summarized in Table 3. After 35 days of growth, no significant difference in plant length was observed between the negative control and the fertilizers, except for AHB+MA 85/15, AHB+MA+PNSB 85/7.5/7.5 and AHB+MA 75/25 (Table 3). Plant length after 45, 55 and 62 days, however, was significantly higher for all fertilized treatments than the negative control. Significant differences in plant length among fertilized treatments were only found after a growing period of 45 days. At 55 and 62 days, all fertilizers performed equally, thereby, showing that the nutrient release pattern of the microbial fertilizers fits the actual need of the plant.

**Table 3:** Pot trial with tomato: Total plant length (mean ± standard deviation per treatment). A non-fertilized negative control (Neg control) was compared to a conventional organic fertilizer (SF2) and three microbial fertilizers, i.e. aerobic heterotrophic bacteria (AHB), microalga (MA) and purple non-sulfur bacteria (PNSB), individually or in blends (according to nitrogen supply).

Treatment code	Total plant length (cm) after							
	35 days		45 days		55 days		62 days	
Neg control	15.7 ± 2.0	a	23.3 ± 2.9	a	39.6 ± 6.1	a	47.3 ± 5.0	a
SF2	15.7 ± 2.0	a	33.3 ± 2.9	b	77.8 ± 7.6	b	98.9 ± 8.0	b
AHB	16.5 ± 2.2	ab	36.2 ± 3.6	bc	77.2 ± 8.3	b	90.1 ± 9.9	b
MA	17.2 ± 2.0	ab	38.2 ± 2.5	c	82.2 ± 7.4	b	96.9 ± 3.3	b
AHB+MA 85/15	17.4 ± 1.7	b	37.9 ± 3.0	c	82.1 ± 7.9	b	94.9 ± 7.8	b
AHB+PNSB 85/15	16.4 ± 2.3	ab	35.8 ± 3.3	bc	79.4 ± 6.4	b	90.7 ± 7.2	b
AHB+MA+PNSB 85/7.5/7.5	17.5 ± 1.5	b	37.6 ± 3.2	c	84.3 ± 2.3	b	96.2 ± 11.3	b
AHB+MA 75/25	17.5 ± 2.3	b	36.7 ± 3.9	bc	80.2 ± 7.5	b	98.1 ± 9.8	b
AHB+PNSB 75/25	16.8 ± 1.8	ab	36.3 ± 3.3	bc	80.5 ± 6.1	b	98.6 ± 2.8	b
AHB+MA+PNSB 75/12.5/12.5	17.1 ± 1.6	ab	36.6 ± 3.6	bc	80.4 ± 6.9	b	95.4 ± 7.6	b
AHB+MA 95/5	16.5 ± 1.6	ab	37.2 ± 3.0	c	79.9 ± 8.8	b	97.4 ± 10.3	b
AHB+PNSB 95/5	16.1 ± 1.2	ab	36.2 ± 2.3	bc	79.8 ± 6.1	b	97.6 ± 0.6	b

Significant differences among treatments are indicated by letter code.

**Fig 4:**
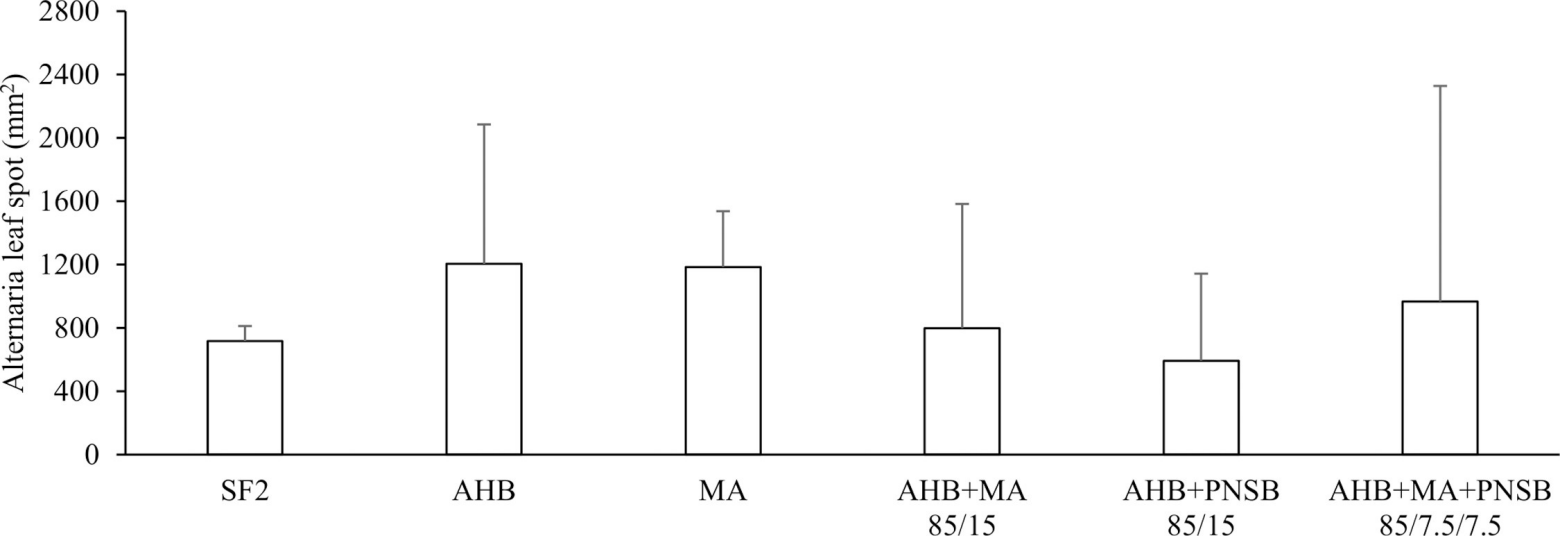
Pot trial with tomato: *Alternaria* leaf spot (mean ± standard deviation) for a selection of treatments. A conventional organic fertilizer (SF2) was compared to three microbial fertilizers, i.e. aerobic heterotrophic bacteria (AHB), microalga (MA) and purple non-sulfur bacterium (PNSB) individually or in blends (according to nitrogen supply). Significant differences among these treatments are indicated by letter code.

Table 4 presents the leaf greenness index, tomato yield and powdery mildew infestation. The leaf greenness index did not vary much among treatments, but the negative control had a significantly lower chlorophyll content than MA, AHB+MA+PNSB 85/7.5/7.5 and AHB+PNSB 75/25. The negative control did not develop quickly enough during the experiment to facilitate tomato harvesting, resulting in significantly higher tomato yield for all fertilized treatments except for AHB+MA 85/15. Susceptibility towards powdery mildew did not differ significantly among treatments. However, the presence of MA in the growing medium significantly reduced powdery mildew infection (p-value 0.000). No significant differences among treatments were observed regarding *Alternaria* leaf spot, nor was there a significant influence of the presence of particular microbial fertilizers (Fig 4). Ammonium toxicity, detectable by leaf chlorosis and stunted growth [41], was not observed for any of the treatments.

**Table 4:** Pot trial with tomato: Leaf greenness index (LGI) after 94 days, tomato yield and disease index upon infection with powdery mildew (mean ± standard deviation per treatment). A non-fertilized negative control (Neg control) was compared to a conventional organic fertilizer (SF2) and three microbial fertilizers, i.e. aerobic heterotrophic bacteria (AHB), microalga (MA) and purple non-sulfur bacteria (PNSB), individually or in blends (according to nitrogen supply).

Treatment code	LGI		Tomato yield		Powdery mildew	
	(SPAD units)		(g plant^-1^)		(disease index)	
Neg control	33.4 ± 3.2	a	0 ± 0	a	n.d.	
SF2	35.7 ± 2.1	ab	576 ± 154	b	1.00 ± 0.00	a
AHB	36.8 ± 2.6	ab	429 ± 237	b	1.00 ± 0.00	a
MA	37.9 ± 3.5	b	516 ± 107	b	0.58 ± 0.49	a
AHB+MA 85/15	34.9 ± 2.9	ab	283 ± 255	ab	0.75 ± 0.27	a
AHB+PNSB 85/15	36.3 ± 3.2	ab	339 ± 184	b	0.83 ± 0.26	a
AHB+MA+PNSB 85/7.5/7.5	37.7 ± 1.0	b	451 ± 98	b	0.75 ± 0.42	a
AHB+MA 75/25	37.5 ± 3.0	ab	459 ± 234	b	0.75 ± 0.27	a
AHB+PNSB 75/25	37.6 ± 2.8	b	545 ± 201	b	1.00 ± 0.00	a
AHB+MA+PNSB 75/12.5/12.5	36.1 ± 3.3	ab	494 ± 183	b	0.75 ± 0.42	a
AHB+MA 95/5	37.3 ± 2.7	ab	627 ± 156	b	0.58 ± 0.49	a
AHB+PNSB 95/5	35.7 ± 2.1	ab	529 ± 165	b	1.00 ± 0.00	a

Significant differences among treatments are indicated by letter code.

This pot trial demonstrated similar plant performance of the microbial fertilizers compared to SF2, with no significant differences in tomato yield. Therefore, it can be stated *a posteriori* that the conventional fertilizer and microbial fertilizers had a nutrient release pattern well aligned to the plant’s needs.

An explanation for the finding that the presence of MA in the growing medium significantly lowered powdery mildew infestation could be attributable to the presence of plant growth-promoting substances (e.g. phytohormones, vitamins and carotenoids; [31, 42, 43]. Several authors have detected phytohormones like indole acetic acid and jasmonic acid in MA, promoting plant growth and supporting plants to cope with biotic as well as abiotic stress [44–49]. Kępczyńska and Król [42] demonstrated that the jasmonic acid derivative methyl jasmonate can elicit induced systemic resistance towards *Alternaria* in tomato. They applied methyl jasmonate by seed soaking or fumigation of seedlings, yet providing this plant hormone using a MA containing fertilizer would be a practical alternative strategy. In a greenhouse trial with tomato, Coppens, Grunert [24] compared three different organic fertilizers incorporated in an organic growing medium: a conventional organic fertilizer and two MA-based organic fertilizers. No significant differences were encountered on plant growth parameters among the three organic fertilizers, yet the MA-based fertilizers significantly reduced tomato yield compared to the conventional organic fertilizer. Fruit quality and thus also market value were, however, enhanced by the application of MA-based fertilizers. Therefore, a tailor-made fertilizer blend would allow combining excellent tomato fruit quality with satisfactory fruit yield, while preserving sustainable cultivation practices (28, 29). Moreover, a gradual increase of the organic nitrogen supply has been demonstrated to improve yields [50]. The use of PNSB as organic fertilizer has also been studied and improved fruit quality [51, 52].

## Conclusions

This study shows that microbial biomass is an excellent means for innovative and sustainable nutrient recycling, yielding microbial fertilizers very well capable of replacing conventional organic fertilizers. Depending on the specific fertilization needs of a particular crop, a tailor-made slow-release organic fertilizer blend can be composed and supplied gradually, maximally aligning with the plant’s needs throughout its growing period. Improved plant product quality and disease resistance can also be accomplished by microbial fertilizers, rendering their application interesting not only in the context of sustainability but also from an economic point of view.

## Supporting information

S1 Table Chemical properties of growing medium. (DOCX)

S1 Data Data manuscript Wambacq & Alloul.xlsx Excel file containing the data of the experiments. (XLSX)
