# Supplementary material for: Aerobes and phototrophs as microbial organic fertilizers: Exploring mineralization, fertilization and plant protection features
Source: PLoS One. 2022 Feb 2;17(2):e0262497. doi: 10.1371/journal.pone.0262497 (PMC8809616; doi:10.1371/journal.pone.0262497)
Supplement: S1 Table — (DOCX) [file pone.0262497.s001.docx]

**S1 Table. Chemical properties of growing medium.**

| **Parameters** |  |  |
| --- | --- | --- |
| pH _(H2O)_ | 6.24 |  |
| EC | 167 | µS/cm |
| NO_3_-N  NH_4_-N  P  K  Ca  Mg  Sulfates  Na  Cl  Fe  Mn | 8.29  11.20  46.30  396  1507  317  103  34.33  119  0.75  3.78 | mg/l  mg/l  mg/l  mg/l  mg/l  mg/l  mg/l  mg/l  mg/l  mg/l  mg/l |
